# Supplementary material for: Dynamic ubiquitination determines transcriptional activity of the plant immune coactivator NPR1
Source: eLife. 2019 Oct 7;8:e47005. doi: 10.7554/eLife.47005 (PMC6850887; doi:10.7554/eLife.47005)
Supplement: Supplementary file 1. [file elife-47005-supp1.docx]

**Supplementary File 1. List of oligonucleotides used.**

| PCR product | Purpose | Sequences (5’-3’) |
| --- | --- | --- |
| *PR1* | qPCR | F - CTAAGGGTTCACAACCAGGC  R - AAGGCCCACCAGAGTGTATG |
| *WRKY18* | qPCR | F - AGAAGGTACAACGCAGCGCAGA  R - TGCGTCCCTTCGTATGTCGCTACA |
| *WRKY38* | qPCR | F - CCGGTTTACCGAACCACTTA  R - GGCTTTCCTTCTCCTGATCC |
| *WRKY62* | qPCR | F - GCCTACACCAAGGACCAGAA  R - AGAGGTGGAGGAGGAGAAGC |
| *NIMIN1* | qPCR | F - CACGGAAACGTAGACGAGAA  R - CCCGTACGACACTGAGAGAA |
| *BIP1* | qPCR | F - CCACCGGCCCCAAGAG  R - GGCGTCCACTTCGAATGTG |
| *PR5* | qPCR | F - ACTGTGGCGGTCTAAG  R – CGTGGGAGGACAAGTTT |
| *TRXh5* | qPCR | F - CATACCCTCGAAGTTTGGAACGAGA  R - TTGCCTCAACTTTGAATTCCTGAGC |
| *PR2* | qPCR | F - CAGATTCCGGTACATCAACG  R - AGTGGTGGTGTCAGTGGCTA |
| *NPR1* | qPCR | F - CTAAAACCGTGGAACTCGGG  R - TCTCTTGTATTTCCATGTACCTTTGCT |
| *PR1* promoter *as1* element | ChIP qPCR | F - AGTGTATACAATGTCAATCGGTGATCTT  R - GCCGCCACATCTATGACGTA |
| *UBP6* | TOPO cloning | F - CACCATGCCTACAGTAAGCGTGAAG  R - TTACATGGAGACGAAGCGGGC |
| *UBP6(C113S)* | Site-directed mutagenesis | F - CTTGGCAACACGTCTTACATGAACTCC  R - GGAGTTCATGTAAGACGTGTTGCCAAG |
| *UBP6* | pET28a cloning | F - GACGAATTCATGCCTACAGTAAGCGTGAAG  R - ACGTGTCGACTTACATGGAGACGAAGC |
| *cul3a* | genotyping | F - CTTAACCGTTTAAAATGGGCC  R - GTCAGATGACGCAGAAAGGAG |
| *cul3b* | genotyping | F - GAGGGAAGACGGTGGAAATAG  R - AAATGCTCCTCCTTGAGCTTC |
| *ube4-2* | genotyping | F - GAACTCGTCTGGTATTTCCCC  R - GAGCTTGCCATGACTTTGAAC |
| *ics1/sid2-2* | genotyping | F - CAATCTTGATGCTCTGCAGCTTC  R - GAAGATAGTTGAACCAAGG |
| *npr1-1* | genotyping (CAPS) | F – CTCGAATGTACATAAGGC  R - CGGTTCTACCTTCCAAAG |
| *uch3-1* | genotyping | F - CGAATCTGATTTTGTGATTCG  R - GAATTTGGTAGGTGCATAGCG |
| *ubp6-1* | genotyping | F – TGGTCCAAGTGGATGGATAAG  R - TGCAAATGGAAGTGGAGAATC |
| *ubp7-1* | genotyping | F – CCCATACTTATGGTGCATCATC  R – AGGCGACAAATATCCAGGTTC |
| *UBE4* | cell-free protein synthesis | F - CCAGCAGGGAGGTACTATGGCGACGAGCAAACCT  R - CCTTATGGCCGGATCCAAGAGCTCTTTTTTTTTTTT  AATCAATTAACATATC |
